# Complex abdominal aortic aneurysms: a review of radiological and clinical assessment, endovascular interventions, and current evidence of management outcomes

**DOI:** 10.1093/bjro/tzae024

**Published:** 2024-08-22

**Authors:** Girija Agarwal, Mohamad Hamady

**Affiliations:** Department of Interventional Radiology, Imperial College Healthcare NHS Trust, London W2 1NY, United Kingdom; Department of Interventional Radiology, Imperial College Healthcare NHS Trust, London W2 1NY, United Kingdom; Department of Surgery and Cancer, Imperial College London, London, United Kingdom

**Keywords:** endovascular aneurysm repair, fenestrated stent, branched stent, chimney, physician modified stent, complex abdominal aortic aneurysm

## Abstract

Endovascular aortic aneurysm repair (EVAR) is an established approach to treating abdominal aortic aneurysms, however, challenges arise when the aneurysm involves visceral branches with insufficient normal segment of the aorta to provide aneurysm seal without excluding those vessels. To overcome this, a range of technological developments and solutions have been proposed including fenestrated, branched, physician-modified stents, and chimney techniques. Understanding the currently available evidence for each option is essential to select the most suitable procedure for each patient. Overall, the evidence for fenestrated endovascular repair is the most comprehensive of these techniques and shows an early post-operative advantage over open surgical repair (OSR) but with a catch-up mortality in the mid-term period. In this review, we will describe these endovascular options, pre- and post-procedure radiological assessment and current evidence of outcomes.

## Introduction

Endovascular aortic aneurysm repair (EVAR) is an established approach to treat aortic aneurysms, being less invasive with lower perioperative mortality, comparable 5-year survival and is preferred by patients.^1^^,^^2^

Challenges arise when the aneurysm involves visceral branches where there is not sufficient normal segment of the aorta to provide an aneurysm seal without excluding those vessels. It has been reported that approximately 30%-40% of infra-renal aneurysms have insufficient neck for a conventional graft or have certain anatomical features that make standard treatment suboptimal.^3^ To overcome the technical challenges, a range of technological developments and solutions have been proposed including fenestrated, branched, physician-modified stents, and chimney techniques with promising outcomes.^4^

Given this challenge of incorporating visceral branches, this review will focus on radiological assessment, device and patient selection and outcomes of endovascular repair for complex abdominal aortic aneurysms.

### Definitions

**Figure 1.**
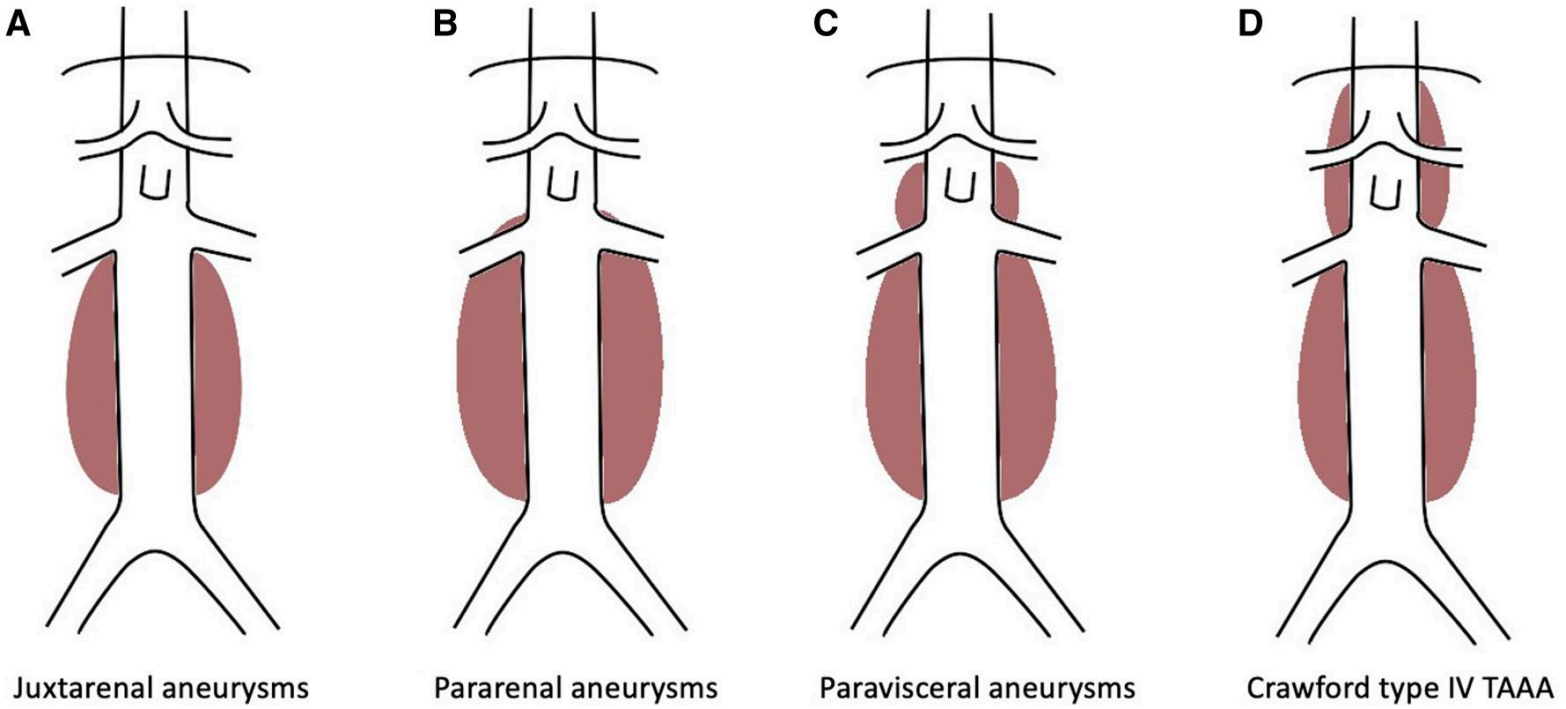
Diagrammatic representation of (A) juxtarenal aneurysms (extends to but does not involve the renal arteries), (B) pararenal aneurysms (aneurysm involves at least 1 renal artery but does not involve the SMA), (C) paravisceral aneurysms (aneurysm involves the SMA but does not involve the coeliac axis), and (D) Crawford type IV thoracoabdominal aneurysms (TAAA). *(Original artwork)*

Complex abdominal aortic aneurysms (Figure 1) include juxtarenal aneurysms (extends to but do not involve the renal arteries), pararenal aneurysms (aneurysm involves at least 1 renal artery and but does not involve the superior mesenteric artery (SMA)), paravisceral aneurysms (aneurysm involves the SMA but does not involve the coeliac axis) and Crawford type IV thoracoabdominal aneurysms (TAAA).^5^

## Indications for intervention

The European Society for Vascular Surgery (ESVS) recommendations for abdominal aorto-iliac artery aneurysms advise considering repair if the aneurysm diameter measures ≥5.5 cm for men and ≥5.0 cm for women, however, these guidelines are for infrarenal AAAs. Data is lacking for complex AAAs though ESVS recommends a similar threshold of 5.5 cm in men and 5 cm in women to consider intervention but this should be evaluated on a case-by-case basis as often these patients are higher surgical risk, meaning a higher diameter may be acceptable.^6^ The NICE guidelines for abdominal aortic aneurysms also advise that symptomatic aneurysms, or those growing at >1 cm/year should be considered for intervention.^7^ For type IV TAA, the ESVS guidelines recommend treatment if the aneurysm diameter measures ≥6 cm, is rapidly expanding (>1 cm/year), or if it is symptomatic; with the caveat that patients with connective tissue disorders may require intervention at lower aneurysm diameter thresholds.^8^

## Pre-intervention assessment

Patient selection is essential to optimise outcomes as having multiple risk factors can make intervention too high risk, while certain comorbidities like peripheral arterial disease, may indicate the patient is more suitable for an open repair.^2^ The new 2024 ESVS guidelines for aorto-iliac aneurysms recommend that for patients with a complex AAA and standard surgical risk, both open or endovascular repair should be considered and the decision should be based on fitness, anatomy, and patient preference. For patients with a complex AAA and high surgical risk, endovascular repair, specifically fenestrated or branched repair, should be considered first line.^6^

The ESVS has comprehensive guidelines on preoperative evaluation of infrarenal AAAs though not so much for juxtarenal aneurysms and variation still exists among different centres in the United Kingdom on the assessment and optimisation of patients.^6^^,^^9^ Nevertheless, it is logical that advice for infrarenal aneurysms can be extrapolated to include complex AAAs. Specific ESVS guidance for juxtarenal AAAs emphasises attempting to preserve renal function as these are closely approximated to the renal vessels and thus the patients at higher risk of renal dysfunction. Preoperatively this can be done through ensuring adequate hydration and holding nephrotoxic medication.^6^ General recommendations are that patients should undergo preoperative cardiac, pulmonary and renal function testing and the risk factors should be optimised.^8^ For example, this can be done by preoperative respiratory training programs for patients with reduced pulmonary function and appropriate medical therapy for blood pressure control. Risk factors can be a predictor of adverse outcomes. Increasing age and renal impairment have been linked to higher post-operative mortality in patients undergoing TAAA repairs, and this should be considered.^10^

### Radiological assessment

Imaging is an integral part of preoperative planning. In terms of imaging technique, CT angiography with 1 mm slices allowing for 3D reconstructions is the standard. Imaging should be interpreted and manipulated using 3D reconstruction software to assess the anatomy, make exact measurements for the stent length and diameter as well as plan the C-arm position and angulation during the procedure. The key anatomical measurements are listed in Table 1. Those measurements are used to assess anatomical suitability for endovascular intervention and to select the appropriate device. Although there is no adverse anatomical feature that represents an absolute contraindication, the increasing number of adverse anatomical features makes the case either substantially difficult or in extreme cases undoable.^8^^,^^11–13^ It is imperative to mention that a CT scan assessment of the arterial anatomy should be complemented by a standard and thorough system assessment to identify any unexpected non-vascular findings.

**Table 1.** Key anatomic measurements for planning complex aortic aneurysm repair.^8^^,^^11–13^

Measurement	Notes	Adverse anatomical feature
**Access vessel morphology (common femoral artery, external iliac artery, and subclavian arteries)**		
Diameter	Minimum and maximum diameter should be recorded	Diameters <7 mm and >23 mm
Calcification	Burden and circumference of calcification in each vessel	Excessive calcification
Mural thrombus	Burden and circumference of thrombus	Thick and circumferential thrombus
Tortuosity	Tortuosity index in external and common iliac arteries	Excessive tortuosity
Length	Length measured along the centreline	<2 cm
Thoracoabdominal angulation	Angulation measured in coronal and sagittal views	>75°
**Proximal landing zone (PLZ)**		
Angulation	Angulation measured in coronal and sagittal views	>65°
Length	Measured at centreline	<2 cm
Diameter	Diameters should be recorded perpendicular to the centreline and along at least the first 2 cm	>31 mm
Shape	Diameter should be taken every 2 mm along the length of PLZ	Conical or reverse conical if oversizing exceeds 20%
Calcification and mural thrombus	The burden and circumference	Excessive calcification or thick thrombus >10 mm
**Visceral segment of the abdominal aorta**		
Aortic diameter at each visceral branch	Measurements taken perpendicular to centreline	Narrow diameter <20 mm and wide diameter >36 mm
Aortic diameter at infrarenal level	Measurements taken perpendicular to centreline	Narrow diameter <18 mm
Aortic diameter at bifurcation	Measurements taken perpendicular to centreline	<20 mm
Aortic thrombus at the level of visceral arteries	Circumference and diameter thickness	Thick and irregular thrombus (shaggy aorta)
Distance from each visceral branch to common iliac bifurcation		
Distance between each visceral artery	Distance taken from the midpoint of each branch to the one below	Distance <5 mm between 2 adjacent vessels
**Side branch information**		
Clock location of visceral vessel origins		
Diameter of each visceral branch	Measurements taken along at the least the first 2 cm	<4 mm and >11 mm
Number and diameter of accessory renal arteries		Accessory and small renal arteries
Stenosis and calcification at the origin of branch vessels		>60 ostial stenosis
Early branch from the visceral artery		Jejunal branch or large polar renal artery branch
Hypogastric arteries	Patency and diameter	Occlusion of hypogastric arteries increases the risk of spinal cord ischaemia or aneurysmal artery >12 mm

## Treatment options

Treatment options include traditional open-surgical repair, endovascular repair, or a hybrid approach. Endovascular techniques include stent graft designs such as fenestrations or branches, to ensure visceral and renal arteries remain perfused. The hybrid approach involves combining surgical and endovascular techniques in which surgical bypasses are created for the branches so the main aneurysm can be excluded endovascularly with a stent.

## Open

Open surgical repair (OSR) is the traditional method of treating complex AAAs but is associated with significant risks. The procedure involves suprarenal aortic cross-clamping which increases the risk of renal impairment, and an estimated 3% of patients go on to require haemodialysis.^14^ Further complications include ischaemic stroke, cardiac complications and death, with a recent 2022 meta-analysis of 22 studies demonstrating a 30-day mortality rate of 4.4% for open repair of juxtarenal AAA.^15^ To overcome this, endovascular and hybrid techniques have been developed which will be discussed in the next sections, however, it is important to note that these too carry a significant burden of risk. For example, after fenestrated endovascular aneurysm repair (fEVAR)/ branched endovascular aneurysm repair (bEVAR), an estimated 2% of patients required new haemodialysis in the perioperative period.^16^

## Endovascular repair

Endovascular stents and techniques have been developed to incorporate the visceral branches and these include grafts with fenestrations or branches and techniques like chimney.

### Fenestrated endovascular aneurysm repair

These fenestrations are holes in the dacron or ePTFE mesh of the stent that can be small, large or semicircular/incomplete holes (scallops), and are reinforced with nitinol wire. These are often custom-made and thus take several weeks to make but can also be off the shelf.^17–19^ In complex AAAs, the proximal landing zone, or even the aneurysm itself can include the visceral branches. Fenestrated stent grafts allow the proximal end of the stent to be positioned at a more appropriate segment of the aorta, thus overcoming this challenge.

There are currently 3 commercially approved devices in Europe which are the Zenith Fenestrated AAA Endovascular Graft by Cook Medical (Bloomington, IN, United States) and Fenestrated Anaconda Custom AAA Stent Graft System (Terumo Aortic, Inchinnan, United Kingdom), and Fenestrated Treo (TerumoAortic, Somerset, NJ, United States), all of which are custom-made. The majority of current research involves the Zenith graft as this is FDA-approved and has been in the market for a long time but its design has several limitations. Specifically, it is less suitable if the branch vessels arise in close proximity to each other and/or the presence of accessory renal arteries; the branches can only be cannulated through femoral access and have no option for partial deployment and adjustment during the procedure. The Anaconda system is a newer device that overcomes some of these limitations, it does not have a metal stent on the graft body which allows versatility of the fenestrations, can be cannulated via upper limb or femoral access, and can also be used for angulated infrarenal necks using the feature of partial deployment and adjustment during the procedure.^20^ Both devices have promising outcomes however a recent review by Jubouri et al^21^ showed that the Fenestrated Anaconda system has a superior performance, with reduced procedural blood loss and lower re-intervention rates when compared to the Zenith graft.^22^

Newer devices are being developed, such as the Zenith p-Branch device by Cook Medical (Bloomington, IN, United States), which is an off-the-shelf device with standard configurations, and thus has the advantage of being readily available rather than needing several weeks for custom production. This device comes in 2 configurations for the renal fenestrations—one with both arising at the same level and the other with the left renal artery offset 4 mm caudally; furthermore, these are adaptive pivot fenestrations which allows for a degree of flexibility. There is also a scallop for the coeliac trunk and a fenestration for the SMA. However, despite these adaptations, less than 50% of patients were found to have anatomy suitable for the Cook p-branch device as per the instructions for use (IFU) criteria in a single-centre study.^23^ The limited evidence regarding this device shows it is acceptable but less favourable in comparison with custom devices. The p-Branch device had higher rates of renal complications, presumably as insertion requires more manipulation during renal artery stenting; and almost 5 times higher rates of re-intervention.^24^

**Figure 2.**
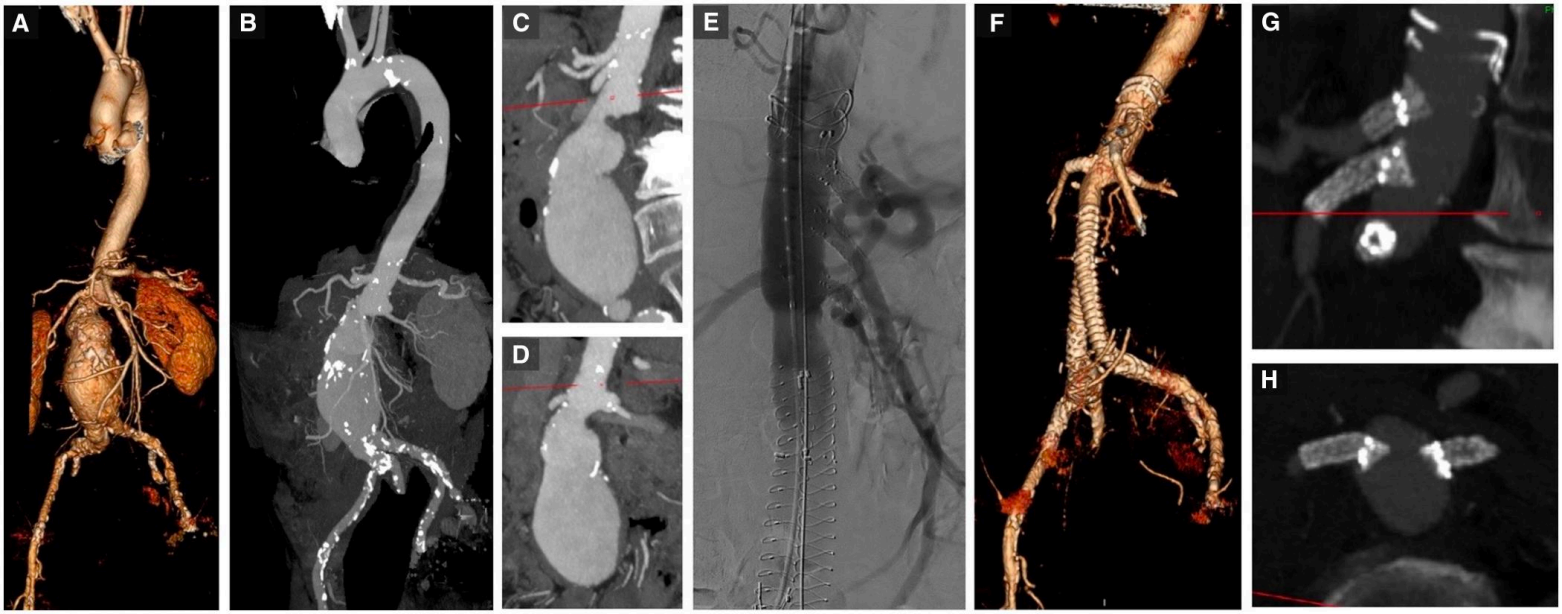
(A-D) A patient with 6.5 cm juxtarenal aneurysm. (E) Fluoroscopic insertion of a custom-made fenestrated Anaconda stent graft with bridging stents into the coeliac, superior mesenteric and both renal arteries. (F-H) Post-operative images demonstrate the fenestrations and bridging stents into the visceral vessels, with the aneurysm satisfactorily excluded.

**Figure 3.**
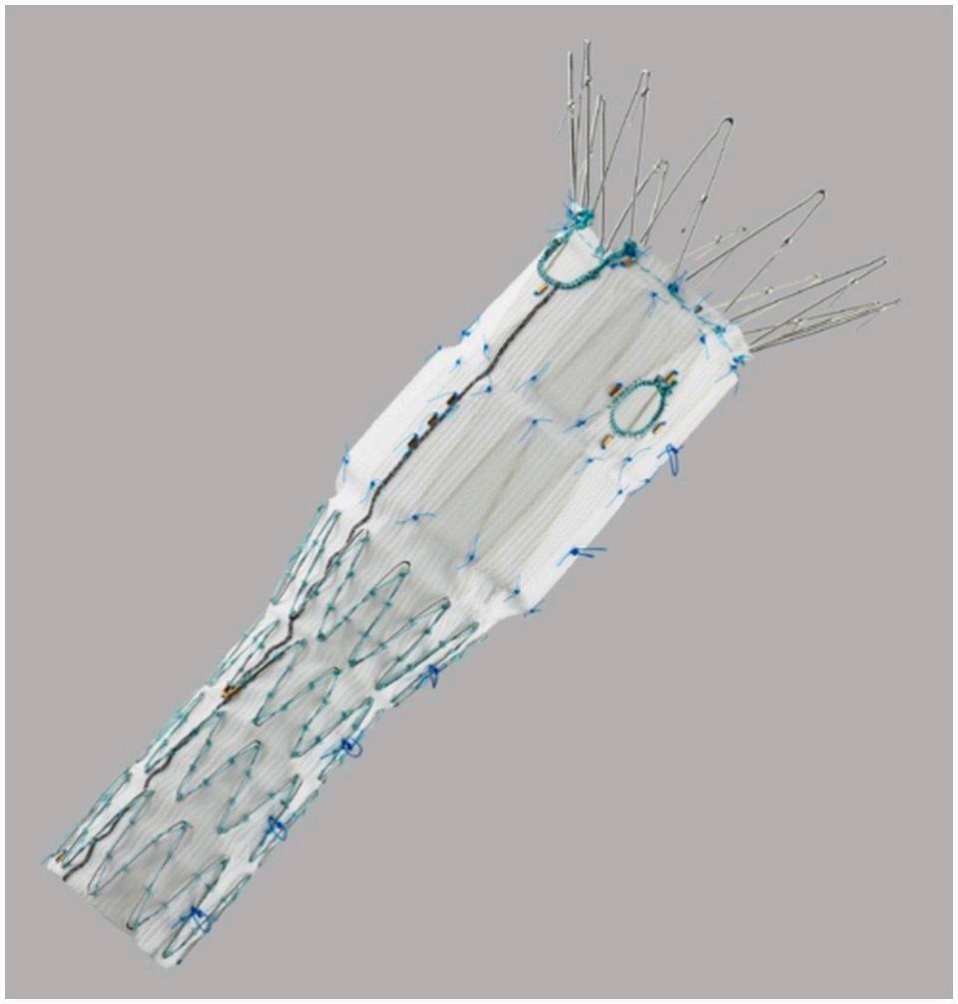
Zenith fenestrated AAA endovascular graft proximal body. Courtesy of Cook Medical.^26^

**Figure 4.**
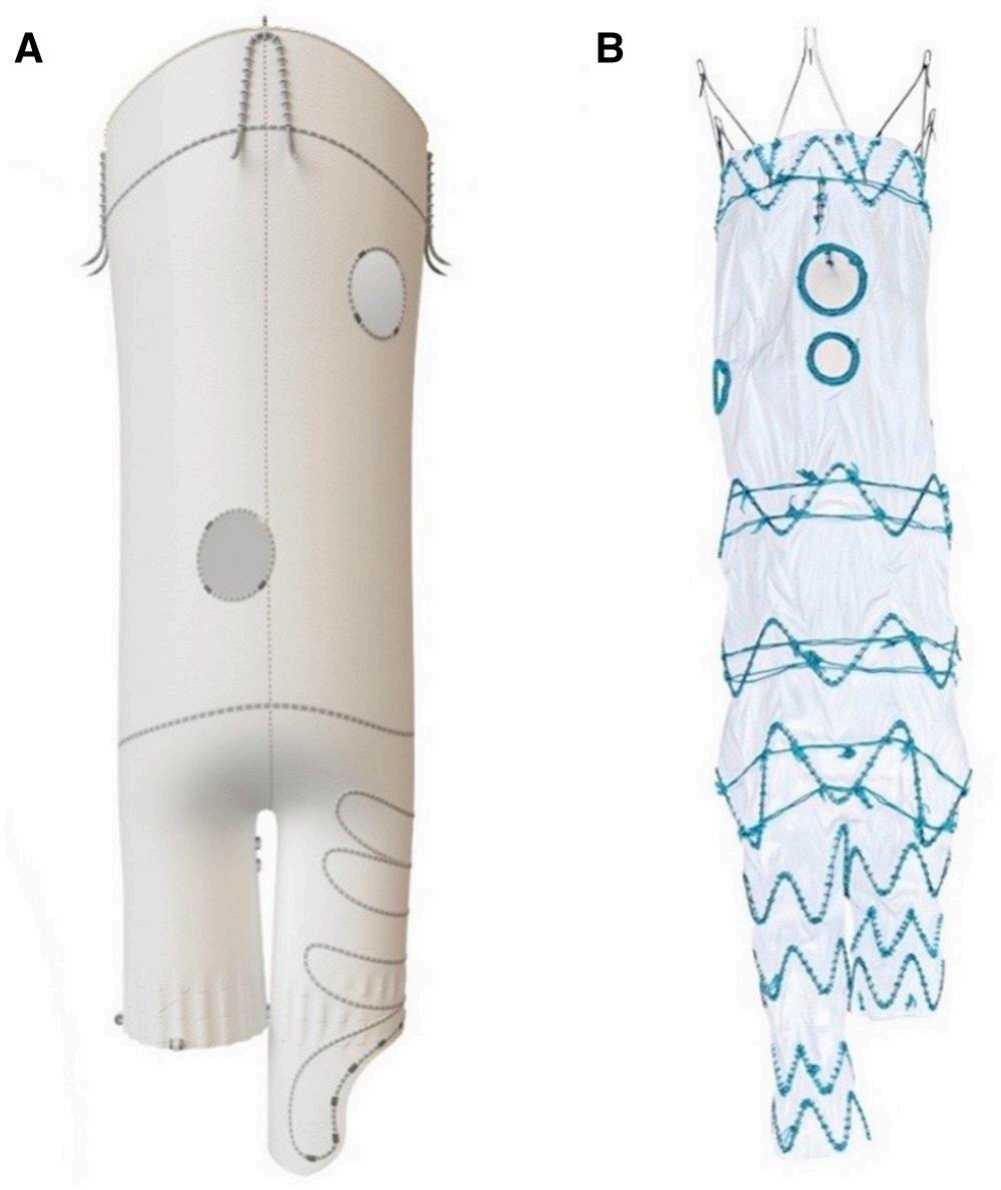
(A) Fenestrated Anaconda custom AAA stent graft system. Courtesy of Terumo Aortic.^27^ (B) Fenestrated Treo device. Courtesy of Terumo Aortic.^28^

Fenestrated grafts are inserted under fluoroscopic guidance, similarly to non-fenestrated grafts. The Anaconda instructions for use recommend the fenestrated graft be oversized by 10%-25%. After insertion, the fenestrations are identified by markers and adjusted to be aligned with the target branches. Once this positioning is satisfactory, the branched vessels can be cannulated and stented under fluoroscopic guidance to create a fenestrated branch stent.^25^ The bridging stents are covered ones and should allow flaring of the aortic side of it. This flaring serves for fixation and sealing. These devices and a case study are shown in Figures 2-4.^26–28^

### Branched endovascular aneurysm repair

Branched stents have downward-facing side branches that are attached to the main body of the stent which improves the seal. These are particularly useful if an aneurysm involves the origin of the visceral branches, or for branches that originate from a large aortic lumen, for example, coeliac and superior mesenteric arteries as well as for thoracoabdominal aneurysms. Some operators also prefer to use this design in cases where there is significant angulation at the level of visceral vessels as it allows more freedom to cannulate the side branches. The stent design can be modified to combine fenestrations and branches to allow better orientation and alignment of the bridging stents, and or facilitate easier cannulation of the side vessel. A covered stent is deployed through the fenestration, into the visceral branch, with its proximal end remaining within the tunnel inside the main aortic stent.^29^

Currently, available devices are off-the-shelf branched stents which include Zenith t-Branch Thoracoabdominal Endovascular Graft (Cook Medical, Bloomington, IN, United States) and E-nside TAAA multibranch stent graft system (Artivion Inc, Kennesaw, GA, United States). These stents have been used for thoracoabdominal aneurysms, including Crawford type IV TAAA. The configuration of all 3 stents is similar, with a main stent body and branched stents to incorporate the 4 visceral arteries (coeliac, SMA and 2 renal arteries), however, they differ in several technical characteristics including length and angulation of the branches. Additionally, the branches in the E-nside system are pre-cannulated inner branches, unlike the TAMBE and t-branch devices which have outer branches. The majority of studies have been done on the t-branch and custom-made Cook stent graft systems and these showed theoretical anatomical feasibility of 32%-88%, which is higher than for TAMBE (30%) and E-nside (43%), though the latter have very few studies. Also in the context of this review, it is important to note these studies included all 4 Crawford type TAAAs, and not just type IV.^30^^,^^31^

**Figure 5.**
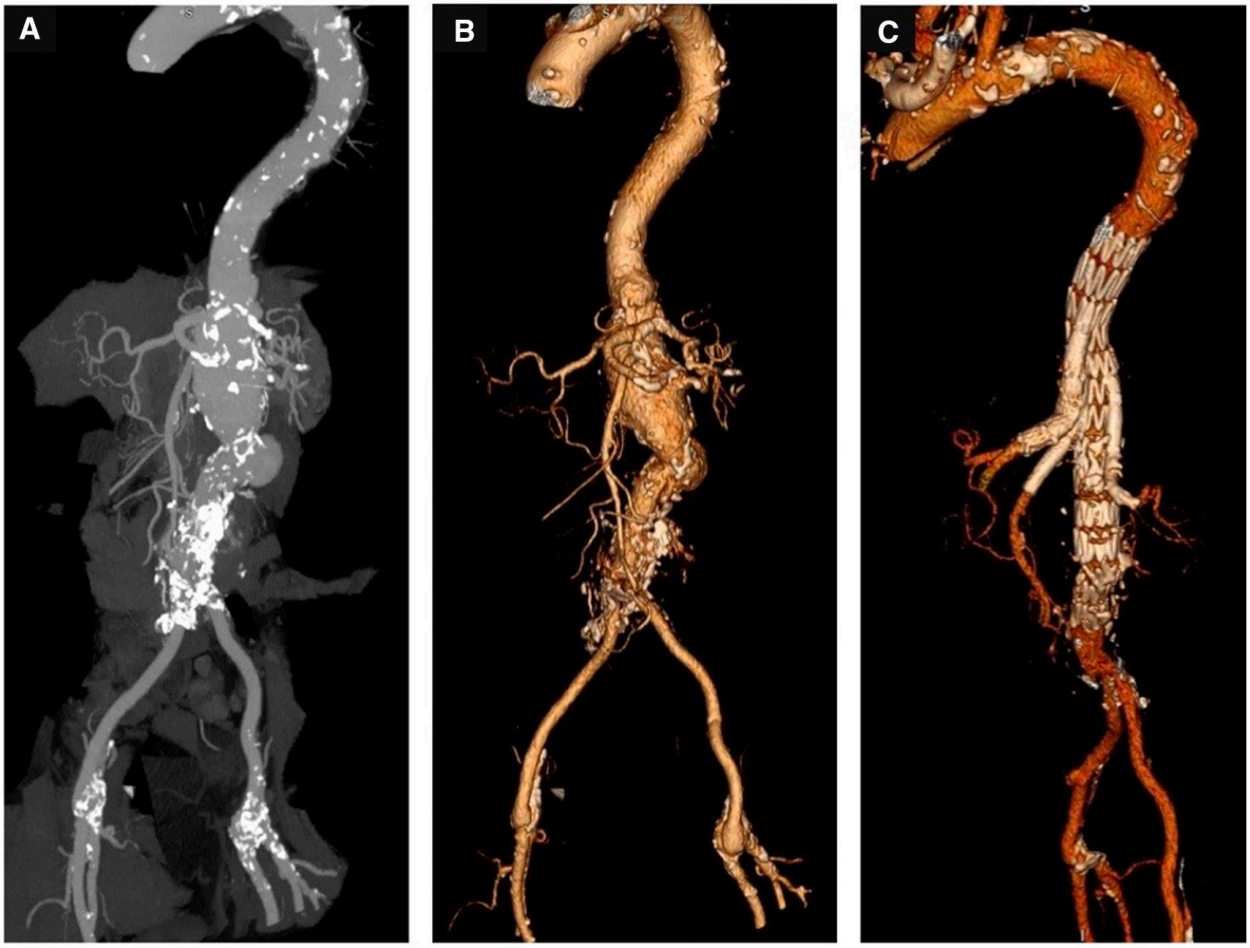
(A, B) Pre-operative imaging demonstrates a Crawford type IV TAAA. (C) The aneurysm was treated with Jotec (Artivion) BEVAR included 3 external branches to the coeliac trunk, SMA, and left renal artery. The aneurysm was satisfactorily excluded.

**Figure 6.**
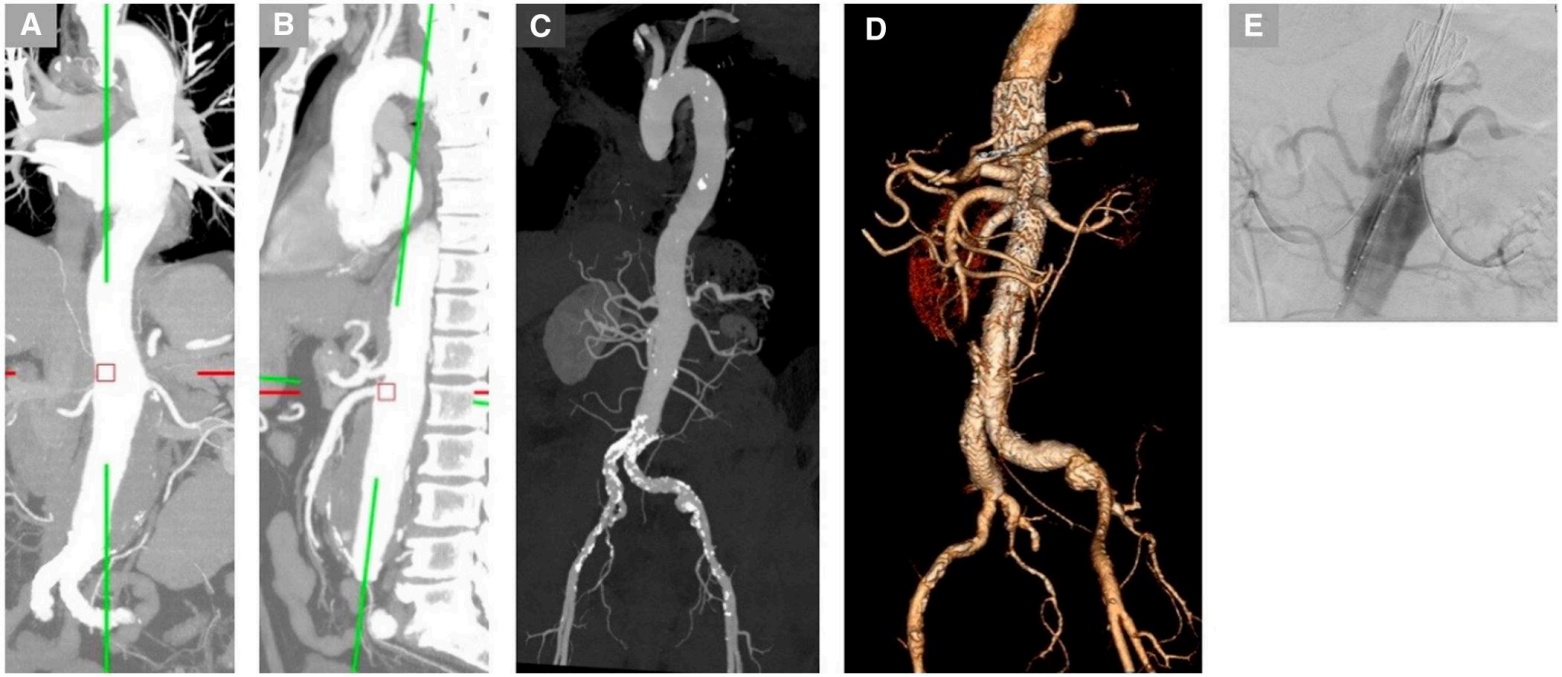
An elderly patient presented with asymptomatic 7 cm juxtarenal AAA. (A-C) Pre-operative imaging demonstrates the aneurysm. (D) The patient was treated with a GORE TAMBE device with 4 internal branches (to the coeliac, superior mesenteric and both renal arteries). Post-operative reconstructed images show the BEVAR stent with branches and excluded aneurysm. (E) Fluoroscopic imaging demonstrates the insertion of the stent.

**Figure 7.**
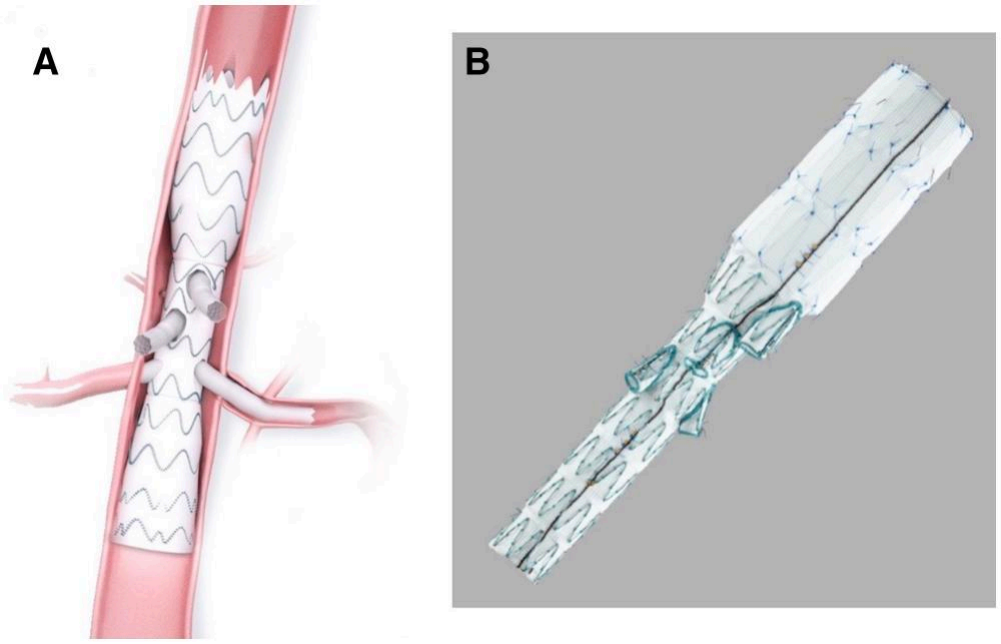
(A) E-nside TAAA multibranch stent graft system. Courtesy of Artivion Inc^34^ (B) Zenith t-Branch thoracoabdominal endovascular graft. Courtesy of Cook Medical.^35^

As with fEVAR, branched stents require femoral access to deploy the main stent device. An antegrade approach with access in the brachial or axillary arteries is usually required to cannulate and bridge the branched grafts. The stent is inserted transfermorally and branches positioned appropriately at the visceral arteries which are then cannulated through the upper extremity access and branched stents deployed. Brachial/axillary access is preferred over contralateral femoral access in these stents owing to their downward-oriented branches, however, with newer technology and steerable sheaths, it may be feasible to perform this retrograde via femoral access.^32^^,^^33^ The devices and case studies are shown in Figures 5-7.^34^^,^^35^

### Chimney endovascular aneurysm repair

**Figure 8.**
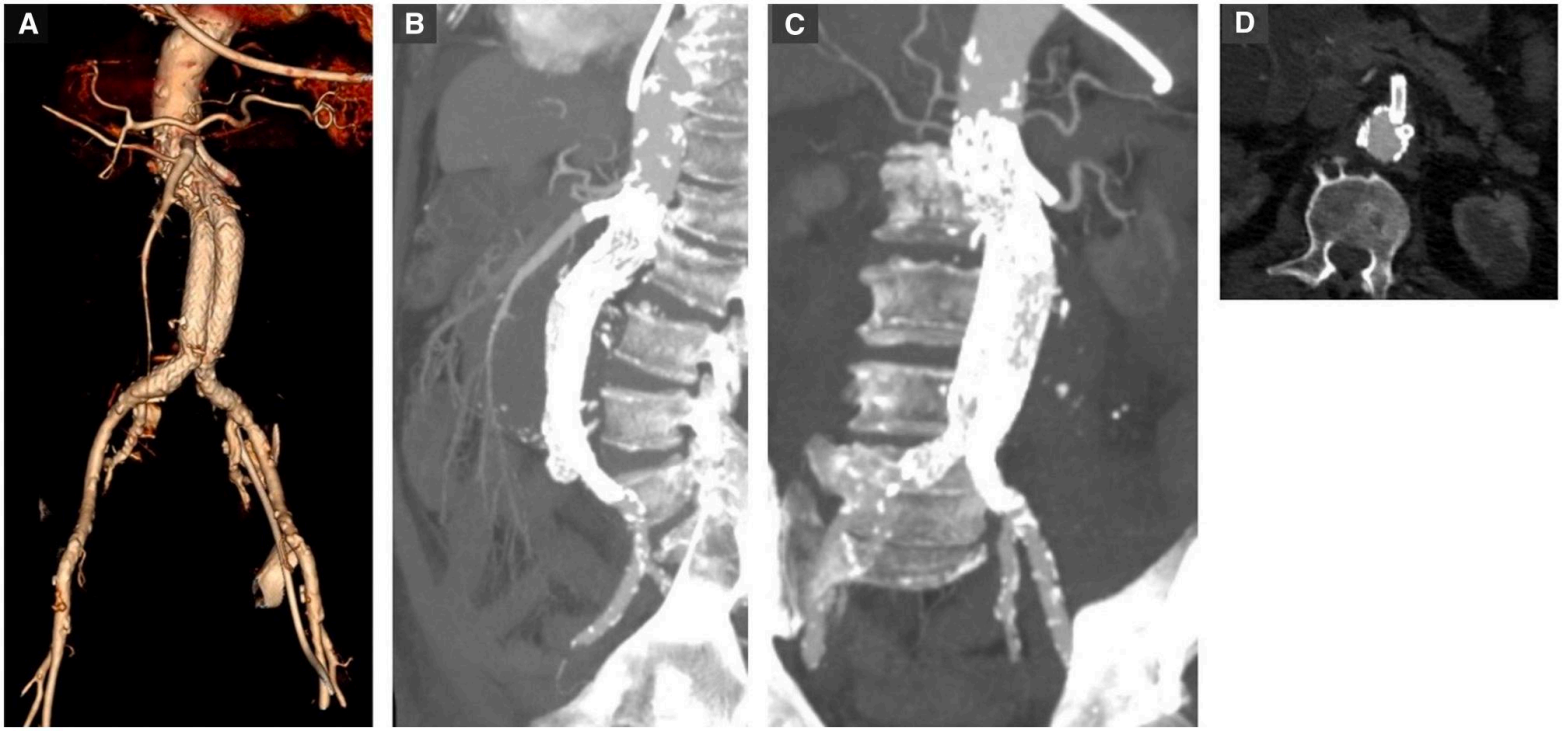
An octogenarian patient presented with an 8 cm abdominal aortic aneurysm and impending rupture. The case was treated with EVAR stent and urgent chimney procedure to the left renal artery and SMA. (A-D) Post-procedure imaging demonstrates the excluded aneurysm and patent visceral arteries.

The Chimney (also known as Snorkel) technique involves placing covered stents into the aortic visceral branches alongside the thoracic or abdominal stent graft to preserve flow to these branches. Similar techniques described include the sandwich and periscope techniques.^36^^,^^37^ In the chimney technique, the proximal extent of the visceral branch graft extends above the aortic graft, whereas in the periscope technique, the proximal end of the visceral graft extends inferior to the distal end of the aortic graft.^38^ These are particularly useful in the time-sensitive urgent cases, as custom-made branched or fenestrated grafts can take several weeks to make.^18^ Importantly, the ESVS guidelines recommend considering the chimney technique only in emergency or bailout cases, and limiting the number of chimneys to ≤2.^6^ Other advantages of the chimney technique over fenestrated grafts include reduced operative time, reduced blood loss and decreased radiation dose.^39^ For patients with narrower vessels, the chimney technique allows the use of standard-sized EVAR devices of 14-18 Fr, as opposed to fenestrated stents which, being more rigid, require larger delivery systems (20-22Fr).^38^ However, a significant limitation is the formation of gutters between the graft and aortic wall, resulting in type I endoleaks.^40^ A case study is shown in Figure 8.

### Physician-modified endografts

Another technique used for more urgent cases is physician-modified stent grafts. Custom fenestrations can be made by the operating physician using cautery and a radio-opaque marker, and often these are used along with covered bridging stents through the fenestration.^41^

There is heterogeneity in the technique used, as there is no agreed standard. In general, the graft is modified on a back table in the operating room after a plan is made preoperatively from imaging. The graft is partially deployed and fenestrations are marked using this plan. With diathermy, wire cutters and cautery the graft and metal stent struts are cut to create fenestrations which can then be reinforced with snares. After this, the graft is resheathed and flushed with an antibiotic such as rifampicin, to minimise contamination.^42^

Newer technologies are being developed and trialled such as using automated planning software and 3D printed models of the patient’s anatomy to assist the physician when creating these fenestrations with promising results.^43^

### Spinal cord ischaemia

Spinal cord ischaemia (SCI) remains an important challenge in both open and endovascular complex aortic surgery. The spinal cord is supplied by not only radicular arteries but also a complex collateral circulation from the subclavian, internal thoracic, lumbar and hypogastric arteries. As a result, SCI is primarily a problem in TAAA repair, and less so in abdominal aortic aneurysms. However, certain complex AAAs like type IV TAAA, those needing extensive aortic coverage, occluded hypogastric arteries or previous aortic surgery are at higher risk of SCI. Since spinal cord perfusion depends on systemic blood pressure and cerebrospinal fluid (CSF) pressure, the risk can be minimised by CSF drainage, maintaining systemic blood pressure, preserving collaterals, staging the procedure and neuromonitoring. ESVS guidelines do not recommend routine CSF drainage in endovascular complex AAA repair but it can be considered in cases with risk factors described above. Another consideration is when using off-the-shelf devices, the extent of aortic coverage should be taken into account to minimise SCI.^6^^,^^44^

### Imaging follow-up and surveillance

There are no clear consensus guidelines regarding surveillance imaging after complex AAA repair, though logically the follow-up would be at least as frequent as after repair for infra-renal AAA and continued long-term. The ESVS guidelines on endovascular repair of juxtarenal AAAs recommend comprehensive follow-up which includes yearly CT angiography.^6^ The IFU for the Zenith Fenestrated Device are thorough and can be extrapolated for most if not all complex AAA repairs. This recommends lifelong surveillance, as with a non-fenestrated device, with additional consideration to evaluate vessel patency at the fenestrations through CT angiography or duplex ultrasound. The specific follow-up protocol includes completion of angiography performed during the procedure, followed by CT angiography at 30 days and then yearly. Optional CT angiography can be performed at 6 months if there are concerning features on the initial 30-day CT.^13^ Unlike conventional EVAR, CT is preferred over duplex ultrasound given that fenestrated/branched grafts are more complex. There is, however, a role for duplex ultrasound to be used as an adjunct to non-enhanced CT for assessing vessel patency in patients for whom contrast is contraindicated. Newer studies show promising results regarding using renal duplex ultrasound for annual surveillance instead of CT but robust evidence is still lacking.^45^

The primary aim of imaging follow-up is to identify endoleaks, monitor sac size and assess device function (migration, component overlap and failure). If complications are noted on CT then further studies such as 3D reconstructions or angiography can be performed. Concerning features may necessitate more frequent imaging follow-up, though local guidelines on this will vary.^6^ The key postoperative imaging findings are listed in Table 2.^13^

**Table 2.** Key post-operative imaging findings.^13^

Findings
Maximum diameter of the aneurysm corrected to the centre-line
Endoleak and type
Stent integrity, including kinking and fractures
Stent position, including stent migration and component overlap
Branch vessel patency

## Evidence

### fEVAR and bEVAR

Unlike infra-renal abdominal aortic aneurysms, the current evidence of comparing endovascular techniques with OSR for complex abdominal aortic aneurysms is limited to observational and population-based studies, with no randomised controlled trials.^46^ This is a challenge as there is often heterogeneity in patient characteristics,^47^^,^^48^ significant variations in reporting standards and sometimes contaminated by industry-sponsored studies with limited quality control. Nevertheless, in a recent meta-analysis by Patel et al^47^ which included 24 studies from 2009 to 2020 and 7854 patients, the reported 30-day perioperative mortality for fEVAR (3.1%) was found to be better than OSR (4.4%). However, mid-term mortality was similar between the 2 groups. Zlatanovic et al, on the other hand, reported midterm mortality, from 16 studies of around 10 years of follow-up in 4369 patients, to be higher in fEVAR (12.3%) when compared with OSR (8.1%), demonstrating a catch-up effect.^49^ Another relevant meta-analysis also by Zlatanovic et al^50^ reported the short-term outcomes of 22 studies and 8853 patients. In these meta-analyses,^47^^,^^49^^,^^50^ the rates of perioperative as well as mid-term renal failure were similar between the fEVAR and OSR groups. The rate of perioperative myocardial infarctions and major adverse cardiovascular events were lower in fEVAR (2.5% and 5%, respectively) compared to OSR (4.2% and 6%, respectively). Regarding rates of re-intervention, perioperatively there was no statistical difference between the groups (7.9% for fEVAR and 9.7% for OSR), but mid-term rates were higher in fEVAR (17.1%) than OSR (3.6%), demonstrating again a catch up effect. There was a higher rate of medium-term visceral branch occlusion/stenosis and spinal cord injuries in fEVAR when compared with OSR. This could be because fEVAR requires a longer proximal landing zone, has a longer operative time, increased blood loss, and more manipulation of the visceral vessels which are all predictors of SCI.

The meta-analysis by Zhou et al^48^ compared fEVAR, bEVAR, chimney endovascular aneurysm repair (chEVAR) and OS and included 25 studies from 2009 to 2021 and 5149 patients with complex AAAs (juxtarenal, pararenal, suprarenal, and TAAA). Though they included studies with type I-III TAAA as well, these were very few and so parameters that included those studies have not been described in this review. They found that fEVAR and OSR had no significant difference in 12- or 24-month branch vessel patency. In terms of re-intervention rates, there was no significant difference at 30 days but at 24 months, FEVAR had higher re-intervention rates than OSR (OR, 2.48; 95% CI, 1.08-5.73). These findings are consistent with those of Patel et al,^47^ Zlatanovic et al,^49^ and Zlatanovic et al.^50^ For the complications of bowel and limb ischaemia, there was no significant difference between fEVAR and OSR.^48^ The evidence for bEVAR alone versus open surgery is scarce as there are only a few relevant studies with a small sample size. All these meta-analyses are limited as evidence for complex AAAs including type IV TAAAs is insufficient since only observational studies have been published.

The most relevant evidence is probably the 2024 COMPASS study which compared short and mid-term outcomes of fEVAR and OSR cohorts.^51^ A total of 1916 propensity-matched patients were included and classified according to aneurysm neck length (juxtarenal: 0-4 mm, short neck: 5-9 mm, and complex neck: ≥10 mm). Further stratification was done into standard and high risk based on the British Aneurysm Repair score for perioperative risk. In the perioperative period, they found lower mortality after fEVAR (2.2%; OR, 0.25; 95% CI, 0.10-0.64; *P* = .001) than OSR (4.5%), lower early complication rate after fEVAR (55.7%; OR, 0.57; 95% CI, 0.43-0.74; *P* < .001) than OSR (62.4%) and shorter hospital stays after fEVAR (4 days) than OSR (7 days). These findings are consistent with existing literature showing more favourable perioperative outcomes after fEVAR than after OSR. Mid-term outcomes were reported for a median follow-up of 3.5 years. During this period, excluding perioperative deaths, the all-cause mortality was higher in fEVAR (hazard ratio(HR), 2.01; 95% CI, 1.46-2.77) than after OSR, showing again the catch effect seen by Zlatanovic et al.^49^ Notably, however, the COMPASS study performed subgroup analysis showing no significant survival difference between fEVAR and OSR for the juxtarenal aneurysm cohort in standard-risk (HR, 1.64; 95% CI, 0.991-2.71; *P* = .054) and high-risk groups (HR, 2.14; 95% CI, 0.95-4.80; *P* = .066). This higher late all-cause mortality for fEVAR was driven predominantly by the standard-risk short neck (HR, 2.83; 95% CI, 1.21-6.64; *P* = .017) and standard-risk complex neck groups (HR, 4.18; 95% CI, 1.80-9.67; *P* = .001). Similarly, overall mortality was greater after fEVAR (HR, 1.4; 95% CI, 1.05-1.87; *P* = .020) than after OSR. However, specifically for aneurysm-related mortality, there was no significant survival difference between the fEVAR (HR, 1.4; 95% CI, 1.05-1.87; *P* = .020) and OSR cohorts. Regarding the rates of secondary intervention, the findings were in line with current data, demonstrating higher mid-term rates after fEVAR (HR, 5.29; 95% CI, 3.36-8.33; *P* < .001) than OSR, despite there being no significant difference in the early phase (*P* = .26).^51^

Overall the results of the COMPASS study showed a significant perioperative mortality and morbidity advantage of FEVAR over OSR which was reversed in the mid-term period. This effect was most marked in the short neck and complex neck groups, while the juxtarenal aneurysm cohort showed equivalent mid-term survival after FEVAR compared to OSR.^51^ Further studies are summarised in Table 3.^16^^,^^52–64^

**Table 3.** Highlighting the main results of some of the major studies on fenestrated/branched endovascular repair of complex abdominal aortic aneurysms.

Authors, year, journal	No. of patients undergoing FEVAR/BEVAR; type of study and aneurysm	Intervention/device used	Main findings	Follow-up
Haulon et al, 2023, Journal of Endovascular Therapy^52^	186 patients Prospective study Complex abdominal aortic aneurysms: Juxtarenal AAA—48.9% Pararenal—15.1% Type IV TAAA—23.1% Other—12.9%	All patients underwent fbEVAR for the treatment of aortic aneurysms Branched and/or fenestrated custom-made device (Cook Medical)—96.2% Zenith Fenestrated (Cook Medical, CE-marked)—3.8%	Technical success—97.8% 30-day outcomes: 30-day mortality—4.3% Renal failure—8.1% Stroke—1.6% Aneurysm rupture—0.5% Endoleak at procedure: Type I proximal—1.1%; Type I distal—1.6% Type II—14% Type III—3.2% Type IV—none Undetermined—0.5% Endoleak at 2 years: Type I proximal—0%; Type I distal—6.5% Type II—15.4% Type III—4.9% Type IV—none Undetermined—0.8%	Mean follow-up was 1.7 ± 0.6 years
Haulon et al, 2023, Journal of Endovascular Therapy^52^	1073 patients Retrospective study Complex abdominal aortic aneurysms. No limits were placed on the type of aortic aneurysm eligible for inclusion in either study.	All patients underwent fbEVAR for the treatment of aortic aneurysms Branched and/or fenestrated custom-made device (Cook Medical)—80.7% Zenith Fenestrated (Cook Medical, CE-marked)—19.3%	Initial success—94.4% 30-day outcomes: 30-day mortality—5.5% Secondary intervention—15.6% Dialysis—1.6% Stroke—0.2% Aneurysm rupture—0.8% Adverse event rate—34.3% (368/1073)	Mean follow-up was 3.2 years
Katsargyris et al, 2023, European Journal of Vascular and Endovascular Surgery^53^	349 patients Retrospective study Short neck AAA—13.5% Juxtarenal AAA—68.8% Suprarenal AAA—17.8%.	Stent grafts were customised based on the Cook Zenith system (William A. Cook Australia, Ltd, Brisbane, Australia) fitting fenestrations and scallops 922 of 1223 vessels were stented with a bridging stent graft	Technical success—98% 30-day mortality rate—0.9% Peri-operative spinal cord ischaemia—0.6% Permanent dialysis—1 patient Occlusion—14 target vessels 47 re-interventions were performed in 38 patients Estimated survival at 5 years—69.3 ± 3.1%. Estimated freedom from aneurysm-related death at 5 years—98.8 ± 0.7%	Mean follow-up was 49.3 ± 32.2 months (range 1-127 months)
Schreuder et al, 2023, Vascular and Endovascular Surgery^54^	172 patients Prospective study Juxtarenal AAA—94.8% Suprarenal AAA—5.2%	Cook Zenith Fenestrated endograft—92.4% Fenestrated Anaconda endograft—7.6%	30-day mortality—1.2% Survival rate at 1 year—90.7% Survival rate at 3 years—75% Survival rate at 5 years—59.9%	Median follow-up was 5.9 years (interquartile range 3.0-8.8)
Dias-Neto et al, 2022, Journal of Vascular Surgery^55^	466 patients Prospective study Complex AAA—138 patients Type IV TAAA—141 patients Type I-III TAAA—187 patients	Patients were treated with an off-the-shelf multibranch stent graft (t-Branch; Cook Medical Inc, Bloomington, IN) or patient-specific manufactured devices (Cook Medical Inc) with any combination of up to 5 fenestrations or directional branches Patient-specific device—88.2% Off-the-shelf t-Branch—11.8%	Early outcomes for complex AAA: 30-day mortality—0.7% Grade 3 spinal cord ischemia—1.4% Early outcomes for type IV TAAA: 30-day mortality—1.4% Grade 3 spinal cord ischemia—1.4%	Mean follow-up was 28 months (95% CI, 25-30 months)
Dossabhoy et al, 2022, Journal of Vascular Surgery^56^	158 patients Retrospective study Complex aortic aneurysms (juxtarenal aneurysms)	All patients had fenestrated endovascular aneurysm repair using the Zenith fenestrated device (Cook Medical, Inc, Bloomington, IN, United States)	30-day mortality—1.9% 51 total re-interventions 31 branch related 13 for endoleaks (4 type Ia, 3 type Ib, 2 type II, 4 type III) 5 limb related 1 access related 1 for bowel ischemia	Mean follow-up was 33.9 ± 24.3 months
Oderich et al, 2021, Annals of Surgery^16^	430 patients Prospective study Pararenal AAA—31% Type IV TAAA—31% Type I-III TAAA—38%	Patients underwent fenestrated and branched endovascular repair. Reinforced fenestrations were the predominant method of incorporation for patients with PRAs (86%) and Type IV TAAAs (87%), and directional branches were used more commonly for Type I-III TAAAs (61%). Pararenal AAA group: Patient-specific devices—99% Off-the-shelf multibranched stent grafts—1% Type IV TAAA group: Patient-specific devices—93% Off-the-shelf multibranched stent-grafts—7%	**30-day outcomes for pararenal AAA:** Any major adverse event—14% 30-day mortality—0.8% Acute kidney injury—11% New onset dialysis—2% Paraplegia—2% Respiratory failure—1% Myocardial infarction—3% Any stroke—1% Any spinal cord ischaemia—2% **30-day outcomes for Type IV TAAA:** Any major adverse event—23% 30-day mortality—1.5% Acute kidney injury—18% New onset dialysis—2% Paraplegia—2% Respiratory failure—3% Myocardial infarction—5% Any stroke—3% Any spinal cord ischaemia—2% Estimated freedom from all-cause mortality at 5 years for Type IV TAAA—60% ± 8% Estimated freedom from all-cause mortality at 5 years for pararenal AAA—66% ± 7% Estimated freedom from secondary intervention at 5 years for Type IV TAAA—70% ± 7% Estimated freedom from secondary intervention at 5 years for pararenal AAA—70% ± 7%	Mean follow-up was 26 ± 20 months (range, 0.1-75.3 months)
Van der Riet et al, 2021, Journal of Endovascular Therapy^57^	194 patients Retrospective study All had pararenal abdominal aortic aneurysms	All patients underwent FEVAR and at least 1 fenestration bridged with Advanta V12 BECS Zenith FSG (Cook Medical Inc, Bloomington, IN, United States)—96% Anaconda FSG (Terumo Aortic, Inchinnan, Scotland, United Kingdom)—4%	Technical success—93% Endoleak at completion angiography—5 patients (4 type Ia; 1 type III) In-hospital mortality—1.5% 30-day mortality—2.6% Estimated patient survival at 3 years—77% (95% CI, 69%-84%) Estimated freedom from all-cause re-intervention at 3 years—70% (95% CI, 61%-78%)	Median follow-up was 24.6 (interquartile range 1.6, 49.9) months.
Locham et al, 2019, Vascular and Endovascular surgery^58^	162 patients Retrospective study Juxtarenal AAA—74.07%, Pararenal AAA—11.11%, Suprarenal AAA—14.81	162 patients had fenestrated endografts	30-day outcomes: Mortality—2.47% Renal complications 1.85% Cardiopulmonary complications—4.94% Bowel ischaemia—1.23% Stroke—0.62% Lower limb ischaemia—0.62% Surgical site infection—1.85%	This study reported 30-day outcomes only
O’Donnell et al, 2019, Journal of Vascular Surgery^59^	880 patients Registry data All patients had complex AAAs (defined as a proximal extent between the top of the coeliac artery and the lowest renal artery, or an aneurysm with a proximal extent below the renal arteries that was repaired with at least 1 scallop, fenestration, branch, or chimney/snorkel into a renal or visceral artery)	880 patients underwent FEVAR/BEVAR	**Perioperative outcomes:** Mortality within 30 days or within the index hospitalization—3.4% Any complication—27% Major adverse cardiac event—6.1% Acute kidney injury—17% End-stage renal disease—1.3% Reintervention—5% Mesenteric/Colonic Ischemia—2.5% Stroke/transient ischaemic attack—0.8% Myocardial infarction—3% 3-year survival: 90%	–
Calster et al, 2018, Journal of Vascular Surgery^60^	468 patients Retrospective study Type IV—V TAAA or pararenal AAA—52.8% Types I-III TAAA—47.2%	All were with custom-made fenestrated or branched devices or with off-the-shelf branched devices (Zenith t-Branch), manufactured by Cook Medical (Bloomington, IN, United States) FEVAR—80.1% BEVAR—9.2% combination F/BEVAR—10.7%	**TAAA types IV, V or PR aneurysm group:** Acute renal failure—9.7% Spinal cord ischaemia—0.04% Estimated freedom from late target vessel occlusion at 5 years—93.12% (88.26-96.15) Estimated survival at 5 years—65.24% (57.44-71.96) **All patients:** Early occlusion—0.7% of the stented target vessels 30-day mortality—4.9% Endoleaks during follow-up—118 patients Type IA, IB, and IC endoleaks—10, 13, and 3 patients, respectively Type II endoleak—91 patients Type III endoleak—26 patients	Median follow-up was 29 months (8.7-51.1 months).
Tinelli et al, 2018, Journal of Vascular Surgery^61^	102 patients Retrospective cohort study analysing prospectively collected data All were pararenal AAAs which include juxtarenal, suprarenal, and type IV TAAAs	All patients had custom-made fenestrated or branched stent grafts from the manufacturer Cook Medical (Bloomington, IN, United States)	**Perioperative follow-up:** 30-day mortality—2.9% Dialysis—4.9% Permanent dialysis—2.9% Cardiac complications—3.9% Early re-intervention—11.8% Pulmonary complications—5.9% Spinal cord ischaemia—0 **Midterm follow-up:** New permanent dialysis—2.9% Mortality—24% Re-interventions—11 patients	Median follow-up was 38.48 months (interquartile range, 46.59 months)
Glebova et al, 2015, Journal of Vascular Surgery^62^	458 patients Retrospective study All patients had abdominal aortic aneurysms	FEVAR—458 patients	30-day postoperative mortality—2.4% 30-day outcomes: Wound complication—3.5% Cardiac complication—2.2% Renal insufficiency—0.4% Dialysis 1.5% Stroke with neurologic deficit—0.9%	–
Martin-Gonzalez et al, 2015, European Journal of Vascular and Endovascular Surgery^63^	225 patients Retrospective study Juxtarenal/pararenal AAA—67.5% Type IV-V TAAA—10.7% Type I-III TAAA—21.8%	All were with fenestrated or branched endografts manufactured by Cook Medical (Bloomington, IN, United States) Fenestrated—187 patients Multibranched—38 patients	30-day mortality rate—6.2% Post-operative acute renal failure—29% Hemodialysis required—5.9% (permanent in 1 patient) 30-day freedom from renal occlusion—99.5% Renal-related endoleak rate during follow-up—3.7%	Median follow-up (95% CI) was 3.1 (2.9-3.3) years
Amiot et al, 2010, European Journal of Vascular and Endovascular Surgery^64^	134 patients Prospective study Juxtarenal AAA—73.9% Suprarenal AAA—20.1% Type IV TAAA—6%	All patients had a fenestrated endograft manufactured by Cook Inc (Bloomington, IN, United States)	**Early follow-up:** 30-day mortality rate—2% Transient dialysis—3% Permanent dialysis—1% Endoleak—12% (16 patients: 3 type I; 12 type II; and 1 type III) **Late follow-up:** Mortality—12 patients Procedure-related re-interventions—12 patients Type I endoleak—1 Type II endoleak—15 Type III endoleak—1	Median follow-up was 15 months (range 2—53 months)

### Chimney endovascular aneurysm repair

The recent systematic reviews and meta-analyses by Patel et al,^47^ and Zlatanovic et al,^49^ described in the previous section also compared chEVAR with OSR for complex abdominal aortic aneurysms (not including type IV thoracoabdominal aneurysms).^50^ The results showed that the perioperative mortality of chEVAR (3.6%-4.8%) was similar to or better than OSR (4.4%-5.5%), and mid-term mortality was similar between the 2 groups. Regarding complications, there was no statistically significant difference between the rates of renal failure for chEVAR (15.7%) and OSR (19.3%). The rate of perioperative myocardial infarctions and major adverse cardiovascular events were lower chEVAR (3.8% and 4.9%, respectively) compared to OSR (4.2% and 6%, respectively). For re-intervention, the perioperative rates were not statistically different between the groups (9.1% for chEVAR and 9.7% for OSR), but mid-term rates were higher in chEVAR (16.1%) than OSR (3.6%). Similarly, there was a higher rate of medium-term visceral branch occlusion/stenosis in chEVAR than OSR (OR, 16.82; 95% CI, 2.79-176.7). However, the authors noted overall low quality studies in 11 out of 22 included studies and significant risk of bias.^49^^,^^50^

The meta-analysis by Zhou et al^48^ as described in the previous section looked at chEVAR versus OSR for complex AAAs including TAAAs. As before, we have only described the parameters that did not include studies of type I-III TAAA. They found that branched vessel patency rates between chEVAR and OSR were not statistically different at 12 months, however at 24 months, chEVAR had lower branch vessel patency rates than OS (OR, 0.09; 95% CI, 0.02-0.48). A similar trend was seen with re-intervention rates—there was no statistical difference at 30 days but at 24 months, chEVAR had higher rates than OS (OR, 3.07; 95% CI, 1.15-8.18). These findings are similar to those seen by Patel et al^47^ and Zlatanovic et al.^49^ As was seen with fEVAR, the rates of bowel and limb ischaemia were similar for chEVAR and OSR.^48^ Further studies are summarised in Table 4.^58^^,^^59^^,^^65–67^

**Table 4.** Highlighting the main results of some of the major studies on chimney/snorkel endovascular repair of complex abdominal aortic aneurysms.

Authors, year, journal	No. of patients undergoing Chimney repair; type of study and aneurysm	Intervention/device used	Main findings	Follow-up
Alfawaz et al, 2021, Annals of Vascular Surgery^65^	79 patients Retrospective study Pararenal AAA—24.0% Type IV TAAA—68.4% Type III TAAA—7.6%	208 parallel grafts were placed Self-expanding covered stents (Viabahn; W.L. Gore) were used for the renal arteries Balloon-expandable covered stents (iCAST; Atrium) were used for the SMA and Coeliac	Technical success—100% Peri-operative mortalities—6 patients End-stage renal disease—2 patients Paraplegia from spinal cord ischaemia—No patients SMA and Coeliac stents patency—100% Renal parallel graft patency—95% Endoleaks on follow-up imaging: Type II—12.6% Type III—none	Median clinical follow-up was 12 months
Touma et al, 2020, European Journal of Vascular and Endovascular Surgery^66^	201 patients underwent complex abdominal aneurysm endovascular repair using ChEVAR. Juxtarenal AAA—46.8% Pararenal AAA—33.3% Type IV TAA—5% Failures of prior repairs—15.1%	343 branch vessels were re-vascularised (270 renal, 59 SMA, 12 coeliac, 2 polar renal arteries). Devices used: Zenith (Cook)—56.2% Endurant (Medtronic)—19.4% C3 (Gore)—17.4% AFX (Endologix)—2.5% Others—4.5%	Intra-operative mortality—2 patients Major endoleaks (type Ia, Ib, and III) on completion angiography—11.9% Intra-operative thrombosis—7.8% Unexpected additional procedures—11.7% 30-day mortality—11.4% Major postoperative complications—30.3% Early re-interventions—14.9% Acute kidney injury—10.9% Permanent dialysis—1.9% Acute embolic stroke—4.5% Endoleaks: Type I proximal endoleak on the first post-operative imaging—11.9% Late type Ia endoleaks—10.1%	Follow-up was 14.7 ± 18 months
Locham et al, 2019, Vascular and Endovascular Surgery^58^	164 patients Retrospective study Juxtarenal AAA—48.78% Pararenal AAA—20.73% Suprarenal AAA—30.49%	164 patients had chimney repair with a main body device other than fenestrated endografts plus renal stents	**30-day outcomes:** Mortality—7.32% Renal complications—6.10% Cardiopulmonary complications—6.10% Bowel ischaemia—3.66% Stroke—1.22% Lower limb ischaemia—1.83% Surgical site infection—1.22%	This study reported 30-day outcomes only
O’Donnell et al, 2019, Journal of Vascular Surgery^59^	260 patients Registry data All patients had complex AAAs (defined as a proximal extent between the top of the celiac artery and the lowest renal artery, or an aneurysm with a proximal extent below the renal arteries that was repaired with at least 1 scallop, fenestration, branch, or chimney/snorkel into a renal or visceral artery)	260 patients underwent chimney/snorkel repair	Perioperative outcomes: Mortality within 30 days or within the index hospitalization—6.1% Any complication—34% Major adverse cardiac event—11.7% Acute kidney injury—19% End-stage renal disease—2.3% Reintervention—7% Mesenteric/Colonic Ischemia—2.3% Stroke/transient ischaemic attack—3.3% Myocardial infarction—5.6% 3-year survival: 85%	–
Donas et al, 2015, Annals of Surgery^67^	517 patients Retrospective study Juxtarenal AAA—69.6% Suprarenal AAA—25.0% Type IV TAAA—5.4%	In all, 898 target vessels were re-vascularised using chimney grafts Mean number of chimney grafts placed was 1.73 per patient (692 renal, 156 SMA, and 50 celiac)	Perioperative technical success—97.1% Persistent type Ia endoleak—2.9% 30-day mortality—4.9% Late mortality at longest follow-up—15.5% Spinal cord ischemia—0% Embolic stroke—1.7% Pneumonia—3.1% Myocardial infarction—2.1% Graft infection—0.2% Acute kidney injury—17.5% Temporary or permanent dialysis—1.5% Late-onset type Ia endoleak detected at 6 months—0.6% Estimated patient survival at 1 year—84.9% (range: 80.1%-88.0%), Estimated patient survival at 3 years—74.9% (range: 56.1%-79.4%)	Mean imaging follow-up was 17.1 ± 8.2 months (range: 1-70 months)

The quality of studies on chEVAR versus OSR is limited by retrospective observational design and a high number of emergent cases that are unsuitable for open surgery, thus increasing the dissimilarity of the patients and furthering the possibility of bias.

### Physician-modified endografts

Physician-modified endografts (PMEG) have shown promising published results for complex AAAs and type IV TAA. In a recent meta-analysis^68^ of 909 patients from 20 studies, the rate of overall technical success was 99.4%, with aortic or aneurysm-related re-interventions being required in 2.2% of patients and no postoperative ruptures reported. Mortality at 30 days was 1.6% but this rose to 10.6% overall mortality. However, major adverse events (defined as death within 30 days, myocardial infarction, respiratory failure, renal failure, bowel ischaemia, major stroke and definitive paraplegia) occurred in 10.8% of patients. It’s important to note that this study was limited as current published studies are entirely retrospective and again of low quality. Further studies are summarised in Table 5.^59^^,^^69–71^

**Table 5.** Highlighting the main results of some of the major studies on physician-modified endograft (PMEG) repair of complex abdominal aortic aneurysms.

Authors, year, journal	No. of patients undergoing PMEG repair; type of study and aneurysm	Intervention/Device used	Main findings	Follow-up
Chait et al, 2023, Journal of Vascular Surgery^69^	156 patients Retrospective study Complex AAA—57% Type IV TAAA—21% Type I-III TAAA—22%	Modifications were created using Zenith TX2 or Alpha thoracic endografts (Cook Medical, Bloomington, IN) with 1-5 reinforced fenestrations, directional branches, or scallops	**30-day and/or in-hospital outcomes for complex AAA:** Mortality—2% Acute kidney injury—12% New onset dialysis 1% Paraplegia—1% Respiratory failure—7% myocardial infarction—6% Major stroke—1% **30-day and/or in-hospital outcomes for TAAA:** Mortality—10% Acute kidney injury—22% New onset dialysis 4% Paraplegia—6% Respiratory failure—9% Myocardial infarction—3% Major stroke—3%	Mean follow-up was 49 ± 38 months (median, 47 months; IQR, 13-76 months).
Chan et al, 2023, Journal of Clinical Medicine^70^	37 patients Retrospective study Juxtarenal AAA—30 patients Type Ia endoleak after previous EVAR—5 patients Pseudoaneurysm after open infrarenal AAA—2 patients	Endurant II stent graft system (Medtronic, Minneapolis, MN, United States) was used to create the fenestrated physician modified endografts	Technical success—87% **Perioperative outcomes:** Endoleak at completion Type I—none Type II—14% Type III—5% Acute kidney injury—5% Stroke/myocardial infarction/respiratory failure/bowel ischaemia—none Reintervention rate 5% **Midterm outcomes:** Target vessel patency at 1 year—96% Type II endoleaks during follow-up—33% Type I or III endoleak during follow-up—none. One-year overall survival was 93%.	Median follow-up was 17.7 months (range 1-47)
O’Donnell et al, 2019, Journal of Vascular Surgery^59^	256 patients Registry data All patients had complex AAAs (defined as a proximal extent between the top of the celiac artery and the lowest renal artery, or an aneurysm with a proximal extent below the renal arteries that was repaired with at least 1 scallop, fenestration, branch, or chimney/snorkel into a renal or visceral artery)	256 patients underwent physician-modified endograft repair	Perioperative outcomes: Mortality within 30 days or within the index hospitalization—2.7% Any complication—29% Major adverse cardiac event—5.4% Acute kidney injury—18% End-stage renal disease—1.4% Reintervention—2.7% Mesenteric/Colonic Ischemia—2.2% Stroke/transient ischaemic attack—0.9% Myocardial infarction—2.7% 3-year survival: 87%	–
Oderich et al, 2019, Journal of Vascular Surgery^71^	145 patients Retrospective study Pararenal AAA—84 patients Type IV TAAA—26 patients Type I-III TAAA—35 patients	Physician-modified endografts were customized on-site under strict sterile technique using the Cook Zenith or TX2 platform (Cook Medical) with 1-5 reinforced fenestrations, scallops, or directional branches.	Early outcomes: Major adverse events for pararenal AAA—42% Major adverse events for type IV TAAA—65% Estimated survival at 1 year For pararenal AAA—85% ± 4% For type IV TAAAs 84% ± 7% Endoleaks: Total 39% Type I—1% Type II—20% Type III—13% Type IV—none	Mean follow-up was 38 ± 26 months

## Gaps in the knowledge

Although there are promising reported outcome results of endovascular management of complex aortic aneurysms, high-quality evidence is still severely lacking. There are no randomised controlled trials nor high-quality registry, and in current published studies reporting is inconsistent. Although propensity-matched studies have been performed, patients who undergo endovascular repair over open are often more frail and co-morbid, which limits the comparability of outcomes. Prospective and randomised controlled trials are essential, and further studies should be done with standardised definitions and guidelines, such as those proposed by the Society of Vascular Surgery.^5^ Furthermore, the median follow-up time in most existing studies is short, with the majority of cited literature publishing only short-term, and occasionally medium-term outcomes. Data on long-term outcomes is important to address this limitation.

## Future developments

Many exciting new technologies are being developed to improve planning, production and insertion of complex aortic stent grafts. With the advent of machine learning, there are now patient-specific simulations being developed to aid in pre-procedural planning and hence reduce delivery time for custom-made fenestrated grafts. Simulations can also be useful in device insertion by predicting graft torsion and displacement of the branch vessels.^72^ Technological refinements that allow lower device profile and development in image guidance techniques are also needed to improve the success rate, reduce radiation dose and shorten procedure time.

## Conclusion

Endovascular aneurysm repair offers a minimally invasive method to treat complex AAAs with promising results, though evidence is lacking to prove the superiority of EVAR over OSR. Nevertheless, endovascular repair is developing rapidly with newer devices and advanced techniques to overcome the challenges of treating these complex aneurysms.
